# Long term follow-up of health-related quality of life in young adults born very preterm or with a very low birth weight

**DOI:** 10.1186/1477-7525-10-49

**Published:** 2012-05-15

**Authors:** Gijsbert Verrips, Leonoor Brouwer, Ton Vogels, Erik Taal, Constance Drossaert, David Feeny, Marieke Verheijden, Pauline Verloove-Vanhorick

**Affiliations:** 1 TNO, Leiden, the Netherlands; 2 Academic Centre Dentistry Amsterdam, University of Amsterdam, Amsterdam, the Netherlands; 3 Intensive Care Department, Erasmus University Medical Center Rotterdam, Rotterdam, the Netherlands; 4 Institute for Behavioral Research, Faculty of Behavioral Sciences, University of Twente, Enschede, the Netherlands; 5 Kaiser Permanente Northwest Center for Health Research, Portland, OR, USA; 6 Health Utilities Incorporated, Dundas, ON, Canada; 7 Leiden University Medical Center, Department of Pediatrics, Leiden, the Netherlands

**Keywords:** Health-related quality of life, Very low birth weight, Follow up

## Abstract

**Background:**

The purpose was, first, to evaluate changes in health-related quality of life (HRQL) in a cohort of very low birth weight (VLBW; <1500 g.) or very preterm (< 32 weeks of gestation) children between ages 14 and 19, and second, to identify correlates of HRQL at age 19.

**Methods:**

HRQL was assessed using the Health Utilities Index Mark 3 (HUI3). In order to explore correlates of HRQL, we performed a hierarchical regression analysis.

**Results:**

Surviving VLBW children (n = 959) from a 1983 Dutch nation-wide cohort were eligible; 630 participated both at age 14 and 19; 54 at age 19 only. The mean HRQL score decreased from 0.87 to 0.86. The HRQL of 45% was stable, 25% were better and 30% were worse. A regression model showed internalizing problems were related most strongly to HRQL.

**Conclusions:**

In the transition from adolescence to young adulthood, HRQL in Dutch VLBW children was stable at the group level but varied at the individual level. HRQL was negatively associated with internalizing problems and also with physical handicaps. Long-term follow-up studies on the impact of VLBW on HRQL are all the more called for, given the growing number of vulnerable infants surviving the neonatal period.

## Background

In the last decade, the number of very low birth weight (VLBW) children in the Netherlands has increased. Given that most determinants of preterm birth remain stable or are increasing in prevalence, this increase is expected to continue [1]. Due to innovative medical technology, perinatal care has improved enormously since the 1970’s, and a growing number of VLBW children now survive the neonatal period. Several studies have indicated that a substantial proportion of VLBW infants are disadvantaged in many physical and psychosocial areas during childhood and adolescence [2-4]. Outcomes such as cerebral palsy (CP), blindness and deafness, cognitive [5] and behavioral [6,7] problems occur more often in VLBW children than in children born at term.

Mortality and morbidity rates are no longer sufficient to evaluate the impact of preterm birth later in life [8]. Broader measures such as Health-Related Quality of Life (HRQL) are needed to understand the significance of impairments and disability for the child [9]. HRQL incorporates the patient’s perspective [10,11] and is often used to assess the impact of preterm birth and to complement clinical measures [12,13]. Longitudinal studies on changes in HRQL in VLBW subjects are sparse, but receive growing attention [14]. The first aim of our study was to evaluate changes in HRQL in VLBW children between the ages of 14 and 19.

One review of young adult outcomes of preterm birth [13] identified several correlates of HRQL, including weight for gestational age [15-17]; demographic and environmental factors such as parental stress [18] and SES [4,19]; physical factors, such as level of handicap [3,4,20,21] and psychological factors such as coping strategies, self-efficacy and internalizing and externalizing behavior [22,23]. The second aim of our study was to evaluate the relative importance of such correlates of HRQL at age 19.

## Material and methods

### Material

**Figure 1:**
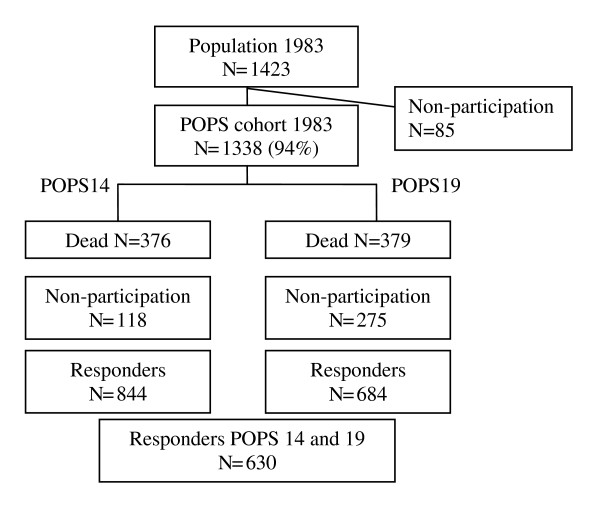
Sampling frame.

Subjects were participants in the Project on Preterm and Small for Gestational Age Infants (POPS), a Dutch nation-wide neonatal follow-up study [15]. POPS was approved by the medical ethics committee of the Leiden University Medical Center. Throughout 1983, POPS enrolled 94% (n = 1338) of all infants in the Netherlands born alive either before 32 completed weeks of gestation, or with a birth weight < 1500 g. Follow-up data were collected at ages two, five, nine, 10, 14 and 19 years. For the purposes of the present study, we mainly used data collected at ages 14 and 19. Participants gave their informed consent prior to inclusion in the study. Figure 1 presents the sampling frame of our study.

In order to evaluate changes in HRQL in VLBW children between ages 14 and 19, we included 630 adolescents who had participated both at ages 14 and 19. In evaluating the relative importance of correlates of HRQL, we included all 684 subjects who had participated at age 19 and for whom data from assessments prior to age 14 were available.

### Data collection

#### HRQL

HRQL was assessed using the Health Utilities Index Mark 3 (HUI3)[24], a comprehensive generic measure encompassing eight attributes of health: vision, hearing, speech, ambulation, dexterity, emotion, cognition and pain. Each attribute has five to six levels of functioning, ranging from level 1 (perfect function) to level 6 (severe dysfunction). The level at which a subject functions with regard to each of the eight attributes is established through questionnaire or interview, which are then used to determine an eight-element health status vector. A utility function may be used to assign a Multi Attribute Utility (MAU) to any particular health status identified [25]. This MAU is a continuous estimate of a population-based preference for a specific health state, yielding an index in which 0 indicates ‘dead’ and 1.0 indicates ‘perfect health’. Also, a Weighted Single Attribute Score (WSAS) may be calculated for each attribute. MAU and WSAS may be categorized into four levels of disability: none, mild, moderate and severe [26,27]. Respondents are the patients themselves, or proxies such as parents. In our study, the primary source of information on HRQL were the adolescents themselves, by questionnaire self-completed at home, at both ages. A number of severely impaired adolescents were unable to provide information. They suffered from major handicaps such as severe CP, mental retardation, blindness, deafness or a combination of these conditions, leading to interference with daily living and thus a life of dependency or institutionalisation. In these children, proxy information obtained by questionnaire from parents or caregivers available at both ages (n = 36) was used. Using the results of a study on method and source effects by Verrips et al [28], HUI3 proxy scores were corrected with a constant calculated on the basis of HUI3 information on children for whom such information was available from both parent and child; this constant comprised the mean difference between child and proxy report.

#### Demographic and environmental factors

SES (low, middle or high) was based on the educational level of the mother. Parental stress was measured at age 14 by administering the short version of the Nijmeegse Ouderlijke Stress Index (NOSIK)[29], a valid and reliable Dutch adaptation of the American Parental Stress Index.

#### Perinatal factors

At birth, using criteria of the Amsterdam growth charts, all infants were classified as appropriate and large for gestational age (AGA/LGA), or small for gestational age (SGA)[15,16].

#### Physical factors

The overall physical outcome at age five was diagnosed by a pediatrician, according to the World Health Organisation (WHO) classification of impairments, disabilities and handicaps. Nowadays the term ‘handicap’ may be obsolete. Back then, in the 80’s of the last century, when the level of disability of our cohort was studied, classification of level of handicap was considered best practice. Three levels of handicap were distinguished: none, minor and major. A handicap was considered minor if it did not seriously interfere with everyday life; and major if it led to a life of dependency or institutionalisation [30]. At age 19, neuro-motor function was assessed by a physician: hand function, quality of walking, coordination, posture and passive muscle tone. A score of 0 was the minimal score, 68 was the highest possible score [31].

#### Psychological factors

All psychological factors in the analysis were assessed at age 19. Self-efficacy was measured using the General Perceived Self-Efficacy Scale; total scores range from 10 (low self-efficacy) to 40 (high self-efficacy)[32]. Coping was assessed using the adolescence version of the Cognitive Emotion Regulation Questionnaire [33] which measures nine adaptive and non-adaptive coping strategies. Information on internalizing and externalizing behavior was gathered by means of the Achenbach Young Adult Self-Report (YASR, 1997 edition), an instrument describing eight different areas of psychological functioning. The YASR measures externalizing problems (e.g., intrusiveness, aggressive behavior, delinquent behavior) and internalizing problems (e.g., anxious and withdrawing behavior).

### Analysis

Differences in background characteristics between participants and non-participants were tested by chi-square tests. The distribution of raw HUI3 scores was calculated by attribute and age. The differences in mean MAU scores by age was tested by a paired T-test and a Pearson correlation coefficient of MAU scores between ages 14 and 19 was calculated. MAU and WSAS scores (X) were categorized into four levels of disability: none (X = 1), mild (1 > X > 0.90), moderate (0.90 > X > 0.70) and severe (X < 0.70). MAU disability categories were cross-tabulated by age. Individual changes in MAU and WSAS categories between ages 14 and 19 were classified into a CHANGE score: 1) *better* (transition to a more favourable category), 2) *stable* (same category) and 3) *worse* (transition to a less favourable category). Subsequently, MAU CHANGE scores were correlated with WSAS CHANGE scores using Kendall’s Tau, a rank-correlation coefficient. This was done in order to evaluate the relative contribution of changes in WSAS to MAU CHANGE.

A hierarchical multiple linear regression analysis was performed to evaluate the amount of HRQL variance at age 19 explained by the putative correlates described above. Continuous MAU was the dependent variable. Demographic and environmental variables were entered in a first step, adding peri- natal factors in step two, physical characteristics in step three, and psychological variables in a final step. A test for multicollinearity of predictors showed the largest correlation coefficient between predictors was 0.46. We collapsed LGA and AGA in our regression analysis for two reasons: we were mainly interested in SGA versus the rest and, moreover, we only had 9 LGA in our cohort. A two-sided significance level of 0.05 was used in all tests.

## Results

Table 1 shows participants were more often female, had less handicaps at age five and had a higher SES than non-participants.

**Table 1:** Characteristics of participants at ages 14 and 19 (n = 630); and non-participants at ages 14 and/or 19

		**Participants n (%)**	**Non-participants n (%)**
Gender *	Male	291 (46)	180 (63)
	female	339 (54)	107 (37)
Gestational age (weeks)	<28	70 (11)	41 (14)
	28–29	64 (10)	33 (12)
	29–30	99 (16)	36 (13)
	30–31	113 (18)	60 (21)
	31–32	113 (18)	42 (15)
	>32	171 (27)	75 (26)
Birth weight (grams)	<=1000	96 (15)	34 (12)
	1001–1250	166 (26)	81 (28)
	1251–1500	237 (38)	118 (41)
	>1500	131 (21)	54 (19)
Appropriate for gestational age	yes	391 (62)	182 (64)
	no	238 (38)	104 (36)
Handicap at age 5 *	None	489 (78)	166 (51)
	Mild	109 (17)	94 (29)
	severe	28 (4)	34 (10)
SES *	Low	216 (34)	152 (57)
	Middle	207 (33)	72 (27)
	high	204 (32)	45 (17)

* p < 0.05, chi-square test.

In total, 162 different HUI3 health states were reported at age 14, and 168 at age 19. The raw HUI3 distributions by attribute and age are presented in Table 2. Only in the vision attribute a change of some substance at the group level was found: 9% of young adults had started to wear glasses.

**Table 2:** Distribution (%) of HUI3 attribute levels ate ages 14 and 19 (n = 630)

	**Vision**		**Hearing**		**Speech**		**Ambulation**		**Dexterity**		**Emotion**		**Cognition**		**Pain**	
Level/age	*14*	*19*	*14*	*19*	*14*	*19*	*14*	*19*	*14*	*19*	*14*	*19*	*14*	*19*	*14*	*19*
*1*	74	65	98	98	79	83	96	97	96	96	70	66	79	74	79	75
*2*	25	35	1	0	15	11	2	1	2	2	28	29	9	5	13	16
*3*	1	0	1	1	6	6	1	0	1	1	2	4	7	13	7	6
*4*	0	0	0	1	0	0	0	1	1	1	0	0	4	5	1	3
*5*	0	0	0	0	0	0	0	0	0	0	0	1	1	3	0	0
*6*	0	0	0	0	-	-	1	1	0	0	-	-	0	0	-	-

-: no level 6 has been defined.

A statistically non-significant decline was found in mean MAU score from 0.87 (sd = 0.18; range = −0.20 to 1) at age 14 to 0.86 (sd = 0.20; range = −0.25 to 1) at age 19. At age 14, the distribution of MAU disability categories was: none (35%), mild (20%), moderate (33%) and severe (12%). At age 19, the distribution of MAU disability categories was: none (28%), mild (34%), moderate (24%) and severe (14%). The mean individual MAU difference between age 14 and 19 was 0.01 (sd = 0.18) and the Pearson correlation coefficient was 0.56. The cross-tabulation of MAU categories by age showed that the majority of subjects (n = 283; 45%) were in the same category at both ages, but a considerable proportion were better off (n = 160; 25%) and an even larger proportion were worse (n = 187; 30%). Table 3 shows the percentual distributions of MAU and WSAS CHANGE categories. Hardly any change was observed in the attributes of hearing, ambulation and dexterity. Compatible with the change in raw scores, a change for the worse was found in the vision attribute. In the psychological attributes of emotion, cognition and pain considerable changes were observed, especially in the pain attribute. Subjects reported more pain at age 19 than at age 14.

**Table 3:** Distribution (%) of MAU and WSAS CHANGE categories (n = 630)

	**MAU**	**Vision**	**Hearing**	**Speech**	**Ambulation**	**Dexterity**	**Emotion**	**Cognition**	**Pain**
Better	25	3	1	13	2	1	20	16	12
Stable	45	86	98	78	97	97	60	65	63
Worse	30	11	1	9	1	2	20	19	25

The correlation coefficients between MAU CHANGE and WSAS CHANGE were: vision 0.22; hearing 0.13; speech 0.36; ambulation 0.13; dexterity 0.04; emotion 0.42; cognition 0.38; pain 0.36. Thus, MAU CHANGE was related to change in the psychological attributes of HUI3 more than to change in the physical ones.

In order to identify correlates of MAU at age 19, four regression models were tested. The results are presented in Table 4. Each block of variables added some proportion to the total variance explained (43%). The psychological variables added the largest amount of variance explained (20%). Internalizing problems were most strongly associated with a low HRQL, followed by level of handicap at age five, neuro-motor score, and non-adaptive coping strategies. Although parental stress was a significant correlate in the first three models, its effect was lower and non-significant when the psychological variables were entered into model.

**Table 4:** **Four models of determinants of health utility at age 19 (n = 684), unstandardized regression coefficient B, 95% confidence interval for B (95% CI) and standardized regression coefficient Beta and amount of variance explained by model R**^**2**^

	**B**	**95% CI**	**Beta**
Model 1: demographics and environment (R^2^ =0.06)			
SES	0.004	−0.017–0.024	0.015
Parental stress *	−0.002	−0.003–0.002	−0.241
Model 2: perinatal data added (R^2^ =0.07)			
SES	0.003	−0.017–0.024	0.013
Parental stress *	−0.002	−0.003–0.002	−0.241
Appropriate for age	−0.025	−0.059–0.010	−0.059
Model 3: physical data added (R^2^ =0.23)			
SES	−0.010	−0.029–0.009	−0.042
Parental stress *	−0.001	−0.002–0.001	−0.140
Appropriate for age	−0.024	−0.056–0.007	−0.058
Neuro-motor *	0.006	0.004–0.007	0.245
Handicap age 5 *	−0.090	−0.123– −0.057	−0.240
Model 4: psychological data added (R^2^ =0.45)			
SES	−-0.009	−0.025–0.008	−0.035
Parental stress	0.001	−0.001–0.000	−0.056
Appropriate for age	−0.013	−0.041–0.015	−0.031
Neuro-motor *	0.004	0.003–0.006	0.195
Handicap age 5 *	−0.094	−0.124– −0.056	−0.252
Internalizing *	−0.009	−0.011– −0.007	−0.349
Externalizing	−0.003	−0.006–0.001	−0.067
Self-efficacy	0.002	0.002–0.005	0.033
Coping adapt.	0.001	0.000–0.001	0.070
Coping non-adapt.	−0.002	−0.004–0.000	−0.070

* = p < 0.05.

## Discussion

HUI3 quantifies disability in eight domains of functioning and also quantifies the preference of the general public for each of the health states defined by the HUI3 system. As HUI3 thus incorporates preferences for health states, we feel this is an appropriate measure of quality of life. Furthermore, use of HUI3 had the great advantage of making our results directly comparable to those reported from other countries, for instance Canada and Germany [34]. We strongly favour standardization of HRQoL measurement, even though other measures might have generated relevant disease-specific information [35]. Respondent burden is also at issue here.

Horsman et al [36] found a 0.03 difference in MAU to be clinically important. Our comparison of HRQL at age 14 and 19 showed a 0.01 MAU difference. Clearly then, at the group level no important changes in HRQL were found in our VLBW subjects. HRQL was fairly high at both ages, and almost similar to results reported for the general US population [37,38] and self-reported HRQL in ELBW young adults in Canada [39,40]. It should be remembered though, that participation was related to SES and to level of handicap at 5 years of age [41]. Our results represented less than half of the original cohort. Our data showed non-participants had lower SES and more handicaps and also that, in participants, these factors were negatively related to HRQoL. We hypothesize we only saw a positive tip of the iceberg in our data, due to loss to follow-up.

Saigal et al [40] found a 0.05 HRQL decrease in ELBW subjects between adolescence and young adulthood. Matched controls showed the same decrease. A decrease in HRQL between age 10 and 40 was also reported by Chen et al. [42] in a study of HRQL among 752 persons born between 1965 and 1975 in the US. Perhaps HRQL decreases between adolescence and young adulthood independently of health conditions, due to the increasingly difficult developmental tasks most young adults are confronted with (e.g. choosing their studies or profession, living on their own, and finding a partner). This is consistent with one Dutch study on the psychological well-being of Dutch adolescents, that tended to decrease gradually in the period from 12 to 23 years of age [40].

These findings from the literature are inconsistent with the results we found in the present study, showing no decrease in HRQL between age 14 and 19 at the group level. Since no matched control data of children born at term were available, we have no way of knowing whether VLBW children differed from children born at term in this respect. One explanation for the fact our findings differed from those reported by Saigal et al [40] may be that they used self-perceived utility, whereas we used a MAU function representing preferences of the general Canadian population. Maybe self-perceived HRQL is more sensitive to change. Futhermore, Saigal’s cohort included ELBW children exclusively, whereas the POPS VLBW cohort included only 15% ELBW children. Maybe ELBW children are more vulnerable in growing up, due to their relatively unfavourable start. Our findings may also be the result of social and cultural factors compensating for perinatal disadvantage. As children grow older, the impact of biological and perinatal risk factors diminishes and demographic and psychological factors have a greater influence on the cognitive performance of LBW and preterm children [3,43]. Indeed, our regression analysis corroborated the importance of psychological factors in HRQL. Furthermore, the wider social policy and cultural context may have an impact on HRQL and well-being of children and young adults. A recent UNICEF report [44] on the well-being of children in 21 rich countries found that the Netherlands ranked first place in the overall educational, social, health wellbeing in children, whereas Canada, for example, ranked 12th.. Thus the general favourable conditions of care for children in the Netherlands may also be reflected in the stable HRQL of our VLBW children [34].

Although HRQL was stable at the group level, our analyses of separate HUI3 attributes showed considerable individual change over time. Was this the result of measurement error or was it true change? Part of the changes observed may be due to random error of measurement. Nevertheless, we do not want to exclude the possibility that clinically important changes in HRQL actually took place, especially in the psychological attributes of HRQL. A considerably proportion of subjects were better off in these attributes, but a comparable proportion were worse. Especially the increased proportion of subjects reporting pain is puzzling and needs further research.

Unlike Hack [13], we found that SES was only weakly related to HRQL at age 19. Sigmond-de Bruin suggested that the lack of influence of SES in our cohort might result not only from the high mean level of the SES in the Netherlands, but also from the country’s high accessibility of care, and its relatively low levels of social and economic inequality [4].

The relationship of AGA to HRQL at age 19 was weak. Since AGA is a strong predictor of several health and psychological outcomes at younger age, the impact of AGA on HRQL may diminish with age [2,4]. However, level of handicap at age five was still a good predictor of HRQL at age 19. Assessment of level of handicap early in life may therefore help parents to understand what HRQoL later in life may be.

The importance of physical problems was underlined by the fact that handicap at age five and neuro-motor problems at age nineteen were both related to HRQL.

As mentioned, Saigal et al [40] found no difference between the mean HRQL of young adults born preterm and that of young adults born at term, and concluded that young adults born with a handicap have adapted to their disabilities and view their lives fairly positively. We found handicap measured at age five and neuro-motor score at age nineteen both to be significantly related to HRQL at age 19. Whereas 68% of the young adults without a handicap reported a high HRQL (MAU > 0.90), only 38% of the young adults with a mild to severe handicap reported a HRQL that high. The high mean score for HRQL might thus be explained not by handicapped young adults having a high HRQL, but by the non-handicapped young adults compensating for their handicapped peers in our cohort, thereby raising the mean HRQL to the same level as that in young adults born at term. Our results do not support the assumption that all young adults with a handicap have learned to cope with their handicaps [40].

Our finding that non-adaptive coping strategies were negatively associated with HRQL is consistent with other studies that found an association between a lower HRQL and non-adaptive coping strategies for various diseases [45-47]. Use of strategies such as self-blame, rumination, catastrophizing and blaming others may lead to a lack of confidence in the ability to cope with health problems. In its turn, this might cause a lower HRQL, consistent with previous reports on the reduced activity that results from non-adaptive coping [33].

Future research must create greater clarity on the relationship between psychological problems and HRQL. For instance, do psychological problems cause lower HRQL, or is it the other way around? If it turns out that such problems have an important effect on the HRQL of young VLBW adults, it might be possible to detect and address such problems early. Physicians may be trained in detecting children with non-adaptive coping styles. Interventions could then be designed to teach these children how to cope adaptively, and thereby to smooth the impact of their handicaps.

## Conclusions

At the group level, no important changes in HRQL were found in our VLBW subjects between ages 14 and 19. HRQL was fairly high at both ages, but non-participants probably had a lower HRQL than participants.

Although HRQL was stable at the group level, our analyses of separate HUI3 attributes showed considerable individual change over time. Clinically important changes in HRQL actually took place, especially in the psychological attributes of HRQL. Especially the increased proportion of subjects reporting pain is puzzling and needs further research.

Non-adaptive coping strategies were negatively associated with HRQL. Future research must create greater clarity on the relationship between psychological problems and HRQL. If it turns out that such problems have an important effect on the HRQL of young VLBW adults, it might be possible to design interventions could then be designed to teach these children how to cope adaptively, and thereby to smooth the impact of their handicaps.

Long term longitudinal studies into quality of life consequences of preterm birth later in life are scarce. Our study clearly showed that it is important to evaluate the impact of pre term birth on quality of life in long-term follow-up studies. Although our subjects had been born 19 years before the assessment we report on here and perinatal treatment has improved considerably in the past decades, our outcomes are very relevant nowadays indeed. Due to the same innovative medical technology, more and more vulnerable ELBW and VLBW children survive the neonatal period at increasingly younger gestational ages, thus leading to roughly similar prevalences of functional limitations, disabilities and handicaps. Our findings are relevant for neonatologists, paediatricians, physicians, psychologists, occupational therapists, physical therapists, teachers and parents in their decision making, treatment, counselling, teaching and helping children growing up. We recorded substantial changes in HRQoL between ages 14 and 19, to the positive and the negative. We recommend to incorporate measures of HRQoL in standard clinical procedures.

## Abbreviations

AGA, Appriopriate for gestational age; ELBW, Extremely low birth weight; HRQL, Health-related quality of life; HUI3, Health utilities index mark 3; LGA, Large for gestational age; MAU, Multi attribute utility; POPS, Project on preterm and small for gestational age infants; SES, Socio-economic status; SGA, Small for gestational age; VLBW, Very low birth weight; WHO, World Health Organisation; WSAS, Weighted single attribute score; YASR, Young adult self-report.

## Competing interest

It should be noted that David Feeny has a proprietary interest in Health Utilities Incorporated, Dundas, Ontario, Canada. HUInc. distributes copyrighted Health Utilities Index (HUI) materials and provides methodological advice on the use of HUI.
